# Inhibitory role of copper and silver nanocomposite on important bacterial and fungal pathogens in rice (*Oryza sativa*)

**DOI:** 10.1038/s41598-023-49918-0

**Published:** 2024-01-20

**Authors:** Arnab Roy Chowdhury, Rishikesh Kumar, Arabinda Mahanty, Koel Mukherjee, Sudhir Kumar, Kishor U. Tribhuvan, Rishav Sheel, Srikanta Lenka, Binay K. Singh, Chirantan Chattopadhyay, T. R. Sharma, Vijai Pal Bhadana, Biplab Sarkar

**Affiliations:** 1 ICAR-National Institute of Secondary Agriculture, Namkum, Ranchi, 834 010 Jharkhand India; 2 ICAR-Indian Institute of Agricultural Biotechnology, GarhkhatangaRanchi, Jharkhand 834 003 India; 3 ICAR-National Rice Research Institute, Cuttack, Odisha 753006 India; 4 Birla Institute of Technology, Mesra, Ranchi, Jharkhand 835215 India

**Keywords:** Nanobiotechnology, Plant sciences, Plant stress responses

## Abstract

Rice (*Oryza sativa*) being among the most important food crops in the world is also susceptible to various bacterial and fungal diseases that are the major stumbling blocks in the way of increased production and productivity. The bacterial leaf blight caused by *Xanthomonas oryzae* pv. *oryzae* and the sheath blight disease caused by *Rhizoctonia solani* are among the most devastating diseases of the rice crop. In spite of the availability of array of chemical control, there are chances of development of resistance. Thus, there is a need for the nanotechnological intervention for management of disease in the form of copper and silver nano-composites. The copper (CuNPs) and silver nanoparticles (AgNPs) were synthesized using green route and characterized using different high throughput techniques, i.e., UV–Vis, FT-IR, DLS, XRD, FE-SEM, TEM. The particle size and zeta potential of synthesized CuNPs and AgNPs were found 273 nm and − 24.2 mV; 95.19 nm and − 25.5 mV respectively. The nanocomposite of CuNPs and AgNPs were prepared having particle size in the range of 375–306 nm with improved stability (zeta potential − 54.7 to − 39.4 mV). The copper and silver nanoparticle composites evaluated against *Xanthomonas oryzae* pv. *oryzae* and *Rhizoctonia solani* were found to have higher antibacterial (inhibition zone 13 mm) and antifungal activities (77%) compared to only the copper nanoparticle (8 mm; 62% respectively). Net house trials of nano-composite formulations against the bacterial blight of rice also corroborated the potential of nanocomposite formulation. In silico studies were carried out selecting two disease-causing proteins, peptide deformylase (*Xanthomonas oryzae*) and pectate lyase (*Rhizoctonia solani*) to perform the molecular docking. Interaction studies indicatedthat both of these proteins generated better complex with CuNPs than AgNPs. The study suggested that the copper and silver nano-composites could be used for developing formulations to control these devastating rice diseases.

## Introduction

Rice is the most important staple food for more than two billion population in the South-East Asian countries and Africa. Various diseases of the rice crop are major causes of yield losses that ultimately adversely affect the annual rice production and productivity^1,2^. Among the different fungal and bacterial diseases of rice, sheath blight and bacterial leaf blight (BLB) are the major diseases that interfere with grain maturity and quality causing yield loss in the range of 10–50% in India as well as at the global level^3^. Sheath blight of rice caused by phytopathogenic fungus, *Rhizoctonia solani* is a major destructive disease of rice responsible for degradation of quality and yield^4^. The chemical control being the major option for disease management and only a handful of fungicides are available for the management of rice sheath blight^5^. For the management of sheath blight disease systemic fungicides like carbendazim, mancozeb, captafol, benomyl and validamycin have been found effective^6,7^. However, indiscriminate use of these fungicides could result to development of resistance in the pathogen^8^. New fungicides are therefore required that are less toxic, more selective, and effective against fungal strains that are resistant to other fungicides. Bacterial blight is caused by bacterial pathogen *Xanthomonas oryzae* pv. *oryzae* which induces yellowing and drying of leaves followed by wilting of rice crop. Bacterial leaf blight is the most serious disease in the earlier stage of rice crop and causes significant yield loss and adversely affects the quality of grains^9^. Traditionally, to manage bacterial leaf blight several bactericidal chemicals such as blasticidin, kasumin, kanamycin and streptomycin have been used for chemical management^10^. The new generation antibiotics like niclosamide have also been reported to mitigate incidence of the bacterial blight in rice crop^11^. The repeated use of antibiotics may lead to emergence of bacterial resistance and become inefficacious to manage the disease^12^. Therefore, it is imperative to explore new and advanced strategies to control the fungal and bacterial pathogens of rice crop for sustainable rice production.

Nanotechnology is the one of the promising strategies that finds its application in agricultural sector in the area of crop production as well as crop protection. Nanoparticle having its unique physio-chemical properties exhibits elevated biological activity in lower doses than their bulk counterpart^13–15^.

Biogenic synthesized silver nanoparticle (AgNPs) has been found to be effective in managing the damage caused by the bacterial leaf blight in rice crop^16^. The AgNPs and graphene oxide nanocomposites were also studied as effective ways to manage the causal pathogen of BLB of rice crop and related microorganisms^17–19^. Recent studies have also demonstrated the extensive antimicrobial activities of silver nanoparticles (AgNPs) against bacteria and fungi due to its multiple modes of inhibitory action^20^.

Copper has also been found to have potential anti-bacterial, antifungal, and anti-biofilm functions^21,22^. The Copper metal is traditionally used as fungicide in many fungicidal formulations like Bordeaux mixture, copper oxychloride, etc. for the management of fungal and bacterial diseases of crops. Most of the copper-based agrochemical formulations contain high doses of copper, which is detrimental to environment and agro-ecosystem^23,24^.

Copper nanoparticles (CuNPs) have recently been reported as an economical alternative to other metal and metal oxide nanoparticles in versatile application areas^25,26^. CuNPs could find its pertinence as competitive substitute of commercial antibacterial agents used in management of bacterial pathogens in the agricultural sector^27,28^. The modes of action of CuNPs explained in the literature are as production of free radicals that cause ‘multiple hit damage’ in cellular system and affect the metabolic pathways of bacterial pathogens^29^. Antifungal efficacy of CuNPs have been reported in the management of fungal diseases of different crops like potato, tomato, chili and water melon caused by pathogens *Fusarium* and *Phytophthora*^30–32^. Recent literature also indicated application of CuNPs for the effective management of plant pathogenic bacteria^33^.

The degradation of plant cell wall is one of the preliminary steps in the pathogenesis process. By weakening the plant cell wall to allow the pathogenic fungus to penetrate and colonize it, pectin degradation by fungal encoded pectinases or pectin degrading enzymes also provides a carbon source for the pathogen's growth and metabolism^34^. The pectate lyase (PL) of *Rhizoctonia solani* is a cell wall degrading enzyme. It triggers the elimination cleavage of de-esterified pectin, a crucial element of plant main cell walls^35^. Through a trans-elimination process, PLs efficiently digest polygalacturonic acid, and the manifestation of enzymatic activity depends on Ca^2+^ ions^36^. According to reports, main source of PL secretion were the plant pathogens and their effect was exhibited due to maceration of plant tissues^37,38^ and PLs have been discovered in various microorganisms, such as *Erwinia aroideae*, *Erwinia chrysanthemi*, *Pseudomonas fluorescens*, *Clostridium multifermenras*, and *Fusarium solani* f. sp^39^. Due to the important role played by the pectin degrading enzyme like pectin lyase, these are being seen as key targets in RNAi or genome editing based development of sheath blight disease resistance in rice^34^. Therefore, we took this protein for the molecular docking analysis.

Similarly, peptide deformylase (PDF) is one of the excellent targets for developing anti-bacterial target^40^. This enzyme catalyzes the removal of the N-formyl group from N-terminal methionine following translation and thus it is necessary for survival of the bacteria^41,42^. Another advantage of selecting PDF as a unique enzyme is it does not show any significant similarity between prokaryotic and organellar PDF ligand binding.

Even though AgNPs has been reported to have antimicrobial and antifungal activities against rice pathogens, it is expensive compared to other metal-based pesticides and thus silver-based anti-pathogenic agents are rarely used in agriculture. In the present investigation, we studied the efficacy of a composite of copper and silver nanoparticles to investigate if the copper-silver combination works better than the copper nanoparticle alone.

## Results

### Synthesis of copper and silver nano-composites

**Figure 1:**
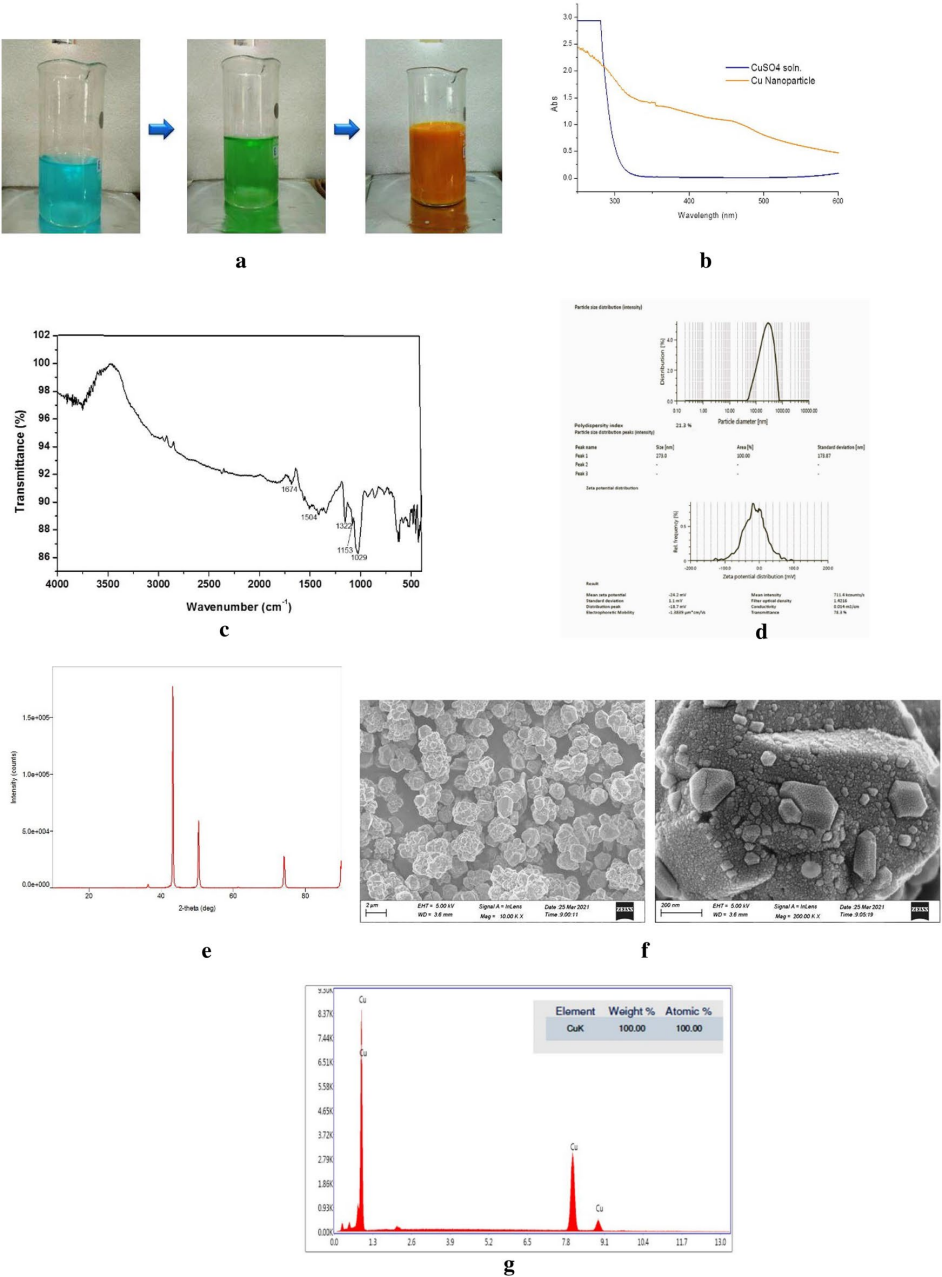
(**a**) Steps for synthesis of CuNPs. (**b**) UV–Vis spectra of synthesized CuNPs. (**c**) FT-IR spectra of CuNPs. (**d**) Particle size distribution and Zeta potential of synthesized CuNPs. (**e**) XRD pattern of CuNPs. (**f**) Field Emission-Scanning Electron Micrograph of CuNPs. (**g**) EDX spectrum of CuNPs.

**Figure 2:**
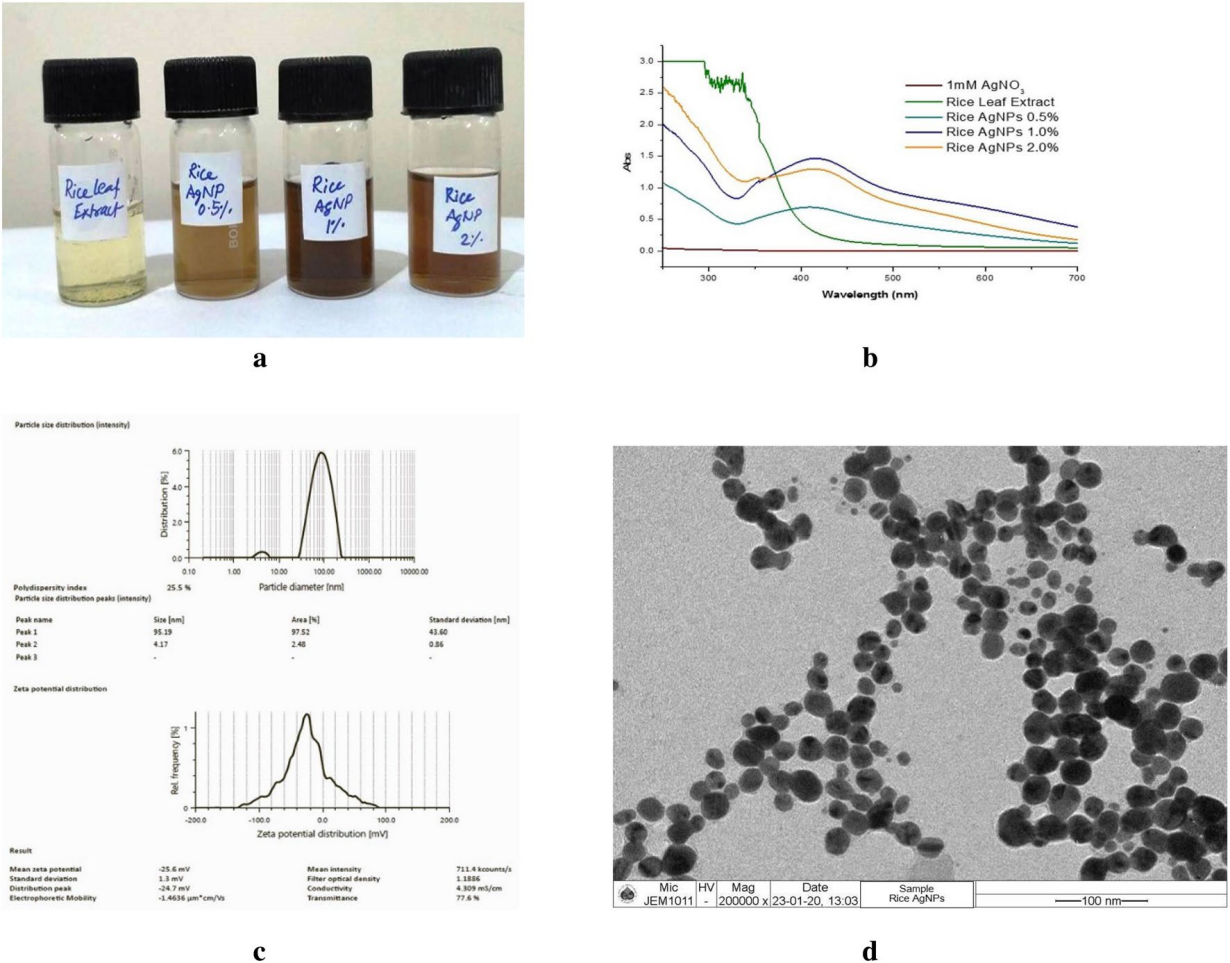
(**a**) Synthesized silver nanoparticles using rice leaf extract. (**b**) UV–Vis spectra of synthesized AgNPs. (**c**) Particle size distribution and zeta potential of synthesized AgNPs. (**d**) TEM images of synthesized AgNPs.

The synthesis of copper nanoparticle was carried out by chemical reduction method and the synthesis was assumed to be completed with the appearance of yellowish-brown color (Fig. 1a). Similarly, the silver nanoparticle was synthesized using green and facile method under ambient temperature using rice leaf extract as biological reduction agent. The formation of silver nanoparticle was confirmed by the appearance of yellow–brown color in the reaction mixture (Fig. 2a).

### Characterization of copper nanoparticles

The synthesized nanoparticles were characterized by a number of techniques like UV–Vis spectroscopy, FT-IR, particle size and zeta potential analysis, XRD and FE–SEM–EDX. The UV–Vis spectra synthesized by copper nanoparticle (Fig. 1b) showed a characteristic peak at 575 nm which confirmed the synthesis of the copper nanoparticle^43^.

FT-IR spectroscopy was employed to look into interaction of various functional groups and species and chemical composition of the organic and inorganic compounds. The peak of the enol hydroxyl and the stretching vibration of the carbon–carbon double bond were both seen at 1674 cm^−1^ and 1322 cm^−1^, respectively which disappeared after the reaction. New peaks were observed at 1504 cm^−1^, 1153 cm^−1^, and 1029 cm^−1^ after the reaction which corresponds to the hydroxyl group, oxidized ester carbonyl group, and conjugated carbonyl group, respectively. According to these findings, surfaces copper nanoparticles contain a polyhydroxyl structure which causes excellent dispersion of copper nanoparticles (Fig. 1c).

The zeta potential and Z-average diameter value of the synthesized CuNPs was found to be − 24.2 mV and 273.0 nm, respectively (Fig. 1d). Polydispersity index (PDI) of the synthesized CuNPs was found to be 21.3%, indicating that the synthesized CuNPs were in mono-dispersed phase with very low chances of aggregation.

The crystal structure and size of the Cu nanoparticles were confirmed by using XRD analysis. The XRD pattern of the ascorbic acid-produced nanoparticles is shown in Fig. 1e. Peaks at 2 values of 36.33, 43.39, 50.49, and 74.18 are associated with the metallic Cu planes (110), (111), (200), and (220). These peaks are highly consistent with the standard JCPDS Card No. 04–0836 for the spectrum of the pure fcc (facial centred cubic) metallic Cu. The mean size of the crystalline Cu nanoparticles calculated from the major diffraction peaks using the Scherrer formula is about 40.11 nm.

The FE-SEM micrograph of the synthesized CuNPs is depicted in Fig. 1f. The size of the copper nanoparticles was determined by FE-SEM which showed that these were below 100 nm and polyhedral in shape. The particles were poly-dispersed in nature and were well distributed. The CuNPs agglomerated to form cluster of aggregated particles.

The EDX spectrum of the synthesized CuNPs was illustrated in Fig. 1g. The EDX spectrum showed two high intensity peaks which confirm the presence of elemental copper in the CuNPs. The EDX spectrum showed the presence of pure elemental copper (100%) and was free of impurities confirming the purity of the synthesized copper nanoparticles.

### Characterization of silver nanoparticles

The synthesized AgNPs were characterized using an array of techniques like UV–Vis spectroscopy, Dynamic Light Scattering (DLS) and Transmission Electron Microscopy (TEM). A broad peak at 415–440 nm was detected in the UV–Vis spectra of the synthesized AgNPs (Fig. 2b) corresponding to the characteristic surface plasmon resonance (SPR) of AgNPs. The Intensity of the peak increased when AgNO_3_ solution reacted with 0.5 percent and 1.0 percent rice leaf extract, however, the intensity decreased with 2 percent concentration of rice leaf extract. Therefore, 1% rice leaf extract was considered as optimum concentration for AgNPs synthesis. Further, no absorption peak corresponding to SPR in respect to controls (rice leaf extract and silver nitrate solutions) was observed. The appearance of the SPR peak in the range of 415–440 nm is a spectroscopic signature to confirm the formation of AgNPs^44^.

The colloidal stability of silver nanoparticles dispersed in the aqueous media and the effective hydrodynamic diameter of the particles was measured via Dynamic Light Scattering (DLS) which determined the particle size by measuring the rate of fluctuations in the laser light intensity scattered by particles as they diffused through solvent. The zeta potential and Z-average diameter value of the synthesized AgNPs was found to be − 25.6 mV and 95.19 nm, respectively. Polydispersity index (PDI) of the synthesized AgNPs was found to be 25.5%, which indicated that the particles were mono-dispersed and there was little probability of agglomeration (Fig. 2c). Poly-dispersity index represents the ratio between different sizes to total number of particles. For the confirmation of size and shape, and morphology of the synthesized AgNPs, TEM study was carried out. TEM image of the synthesized *piyar* gum induced AgNPs showed particle size ranging 13.62–23.34 nm at 200,000× magnification, whereas the shape was spherical in nature (Fig. 2d). TEM results confirmed that AgNPs did not agglomerate, dispersed well and were almost spherical in shape with particle size in nano range. The size of the AgNPs obtained in the current study was matched with earlier reports on rice leaf extract mediated AgNPs synthesis^45,46^.

### Characterization of prepared nanocomposites of CuNPs and AgNPs

**Figure 3:**
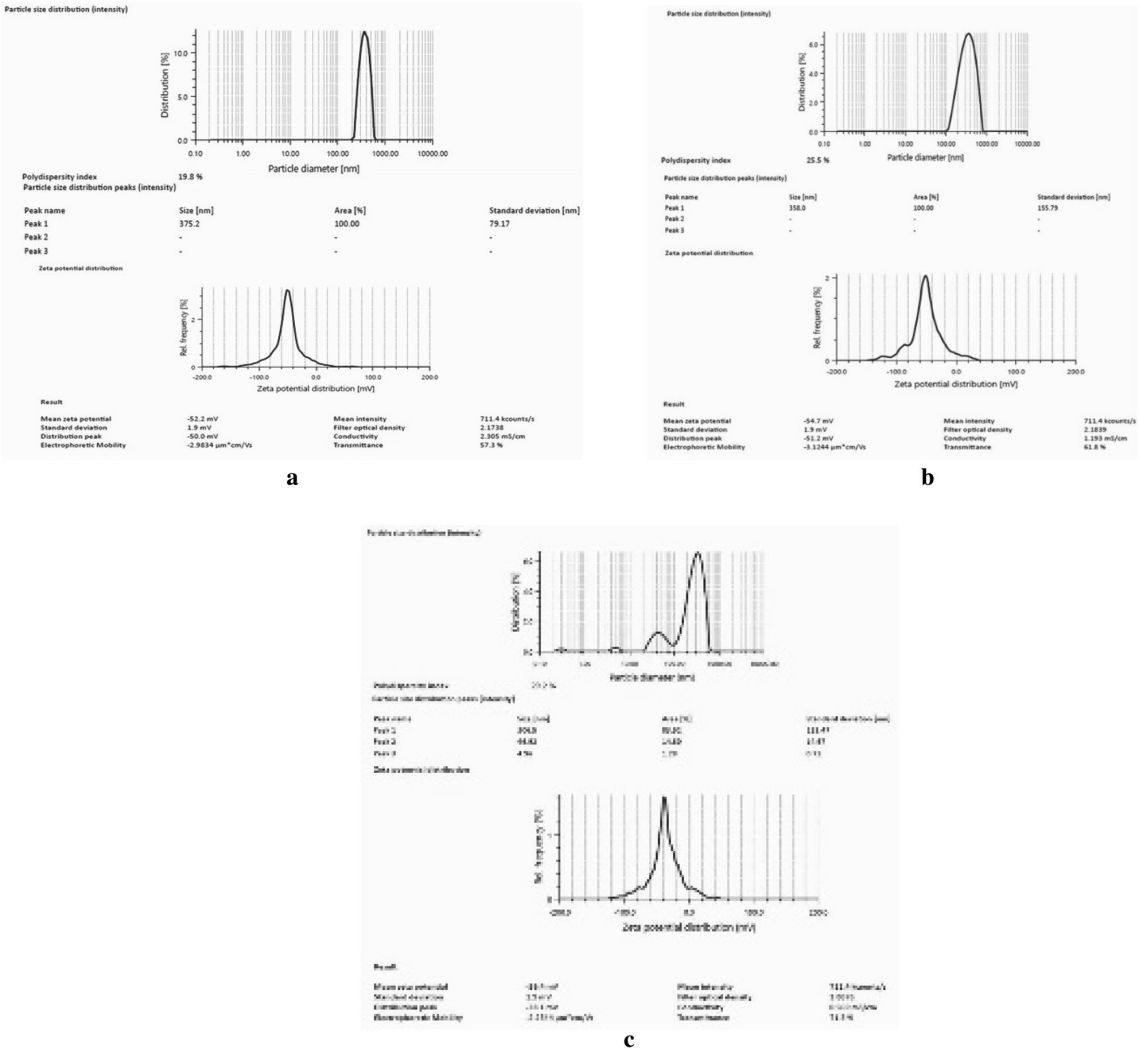
(**a**) Particle size distribution and Zeta potential of Cu and Ag nano-composite (500 ppm CuNPs + 50 ppm AgNPs). (**b**) Particle size distribution and Zeta potential of Cu and Ag nano-composite (250 ppm CuNPs + 25 ppm AgNPs). (**c**) Particle size distribution and Zeta potential of Cu and Ag nano-composite (100 ppm CuNPs + 10 ppm AgNPs.

The particle size, zeta potential and polydispersity index of the prepared nanocomposite of CuNps and AgNPs were measured. The Z-average diameter, PDI and zeta potential of nanocomposite of 500 ppm CuNPs and 50 ppm AgNPs were found to be 375.2 nm, 19.8% and − 52.2 mV (Fig. 3a). The particle size, PDI and zeta potential of nanocomposite of 250 ppm CuNPs and 25 ppm AgNPs were found to be 358.0 nm, 25.5% and − 54.7 mV (Fig. 3b). The 100 ppm CuNPs and 10 ppm AgNPs nanocomposite exhibited the 306.5 nm Z-average diameter, 29.2% PDI and zeta potential of − 39.4 mV (Fig. 3c).

### In vitro antibacterial assay of the CuNPs–AgNPs composites against *X. oryzae*

**Figure 4:**
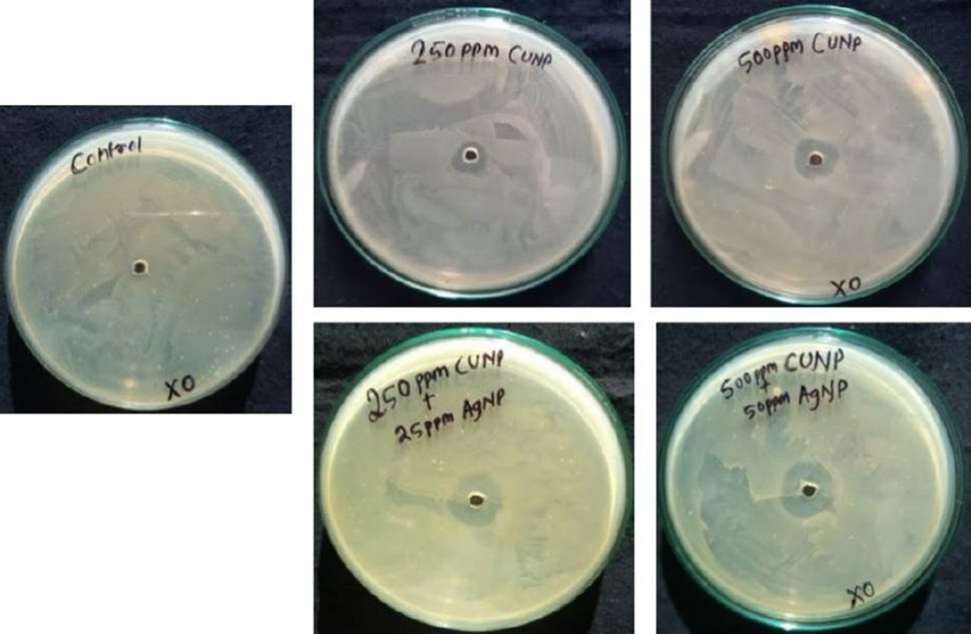
Antimicrobial activity of Cu and Ag nano-composite against *Xanthomonas oryzae*.

The antibacterial activity of CuNPs and CuNPs–AgNPs composites were tested against *X. oryzae* pv. *oryzae* at different concentrations. The diameters of zone of inhibition were 8 ± 0.7 mm and 11 ± 0.7 mm when 250 and 500 ppm of CuNPs were used individually. Apart from this, lower doses of CuNPs viz., 100 ppm and 50 ppm were also tested; however, we could not detect any zone of inhibition at these concentrations indicating that 250 ppm of CuNPs could be the effective dose for in vitro antibacterial assay against *X. oryzae* pv*. oryzae.* The diameters of zones of inhibitions increased when CuNPs-AgNPs composites in comparison to when the same concentration of CuNP was used individually indicating the synergistic effect of the CuNPs and AgNPs (Fig. 4, Table 1).

**Table 1:** Antimicrobial activity of nano-composite against *Xanthomonas oryzae* pv. *oryzae.*

Concentration of nano-composite	Diameter (zone of inhibition) in mm
Control	0.0^a^
250 ppm CuNP	8.3 ± 0.57^b^
250 ppm CuNP + 25 ppm AgNP	13 ± 1^c^
500 ppm CuNP	11.66 ± 0.57^d^
500 ppm CuNP + 50 ppm AgNP	16.3 ± 0.57^e^

Different letters in superscript indicate significant difference (p < 0.05).

### In vitro antifungal assay of the CuNPs–AgNPs composite against *R. solani*

**Figure 5:**
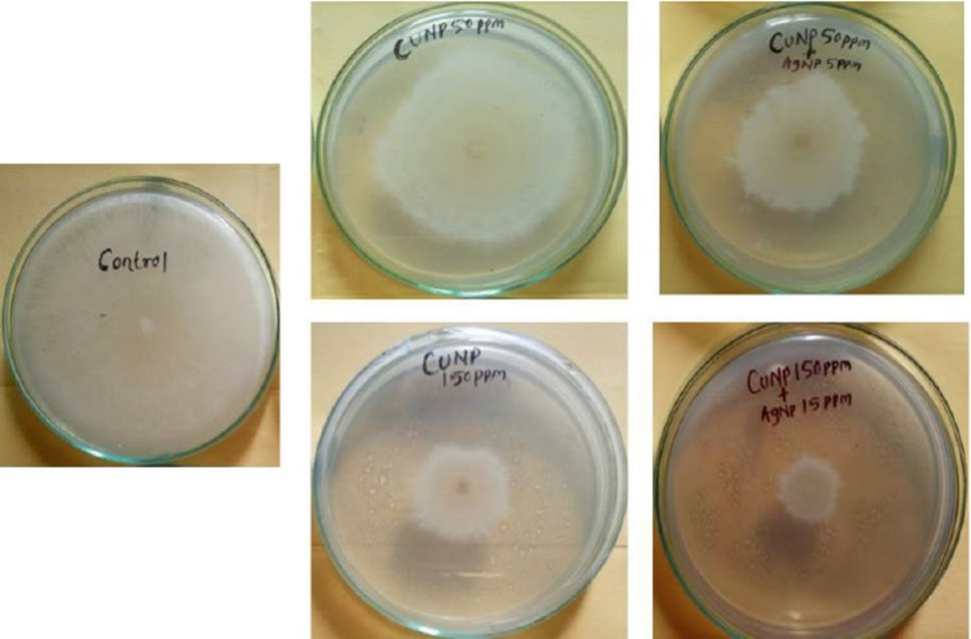
Antifungal activity of Cu and Ag nano-composite against *Rhizoctonia solani*.

In vitro antifungal assay showed that both the CuNPs and CuNPs–AgNPs composites were found to be inhibiting the growth of the fungal pathogen *Rhizoctonia solani* (Fig. 5, Table 2). The % of growth inhibition increased with increase in the concentration. Similar to the antibacterial activity, the CuNPs-AgNPs composite was found to be more effective than the CuNPs used singly. At 150 ppm the CuNPs could inhibit the growth of *R. solani* by 62% whereas CuNPs-AgNPs (150 ppm + 15 ppm) composites could reduce the growth by 77% indicating the high antifungal activity of the CuNPs-AgNPs composite (Fig. 5, Table 2).

**Table 2:** Antifungal activity of the nano-composite formulation against *Rhizoctonia solani*.

Conc of nano-composite	% growth inhibition of *Rhizoctonia solani* in comparison to control
50 ppm CuNP + 5 ppm AgNP	37.05 ± 0.58^a^
50 ppm CuNP	28.23 ± 0.52^b^
100 ppm CuNP + 10 ppm AgNP	66.47 ± 0.56^c^
100 ppm CuNP	57.64 ± 0.58^d^
150 ppm CuNP + 15 ppm AgNP	77.05 ± 0.54^e^
150 ppm CuNP	62.64 ± 0.41^f^

*Different letters in superscript indicate significant difference (*p* < 0.05).

### Net house trials of copper and silver nano-composite formulations *against Xanthomonas oryzae *pv. *oryzae* in cv. TN-1 of rice

**Figure 6:**
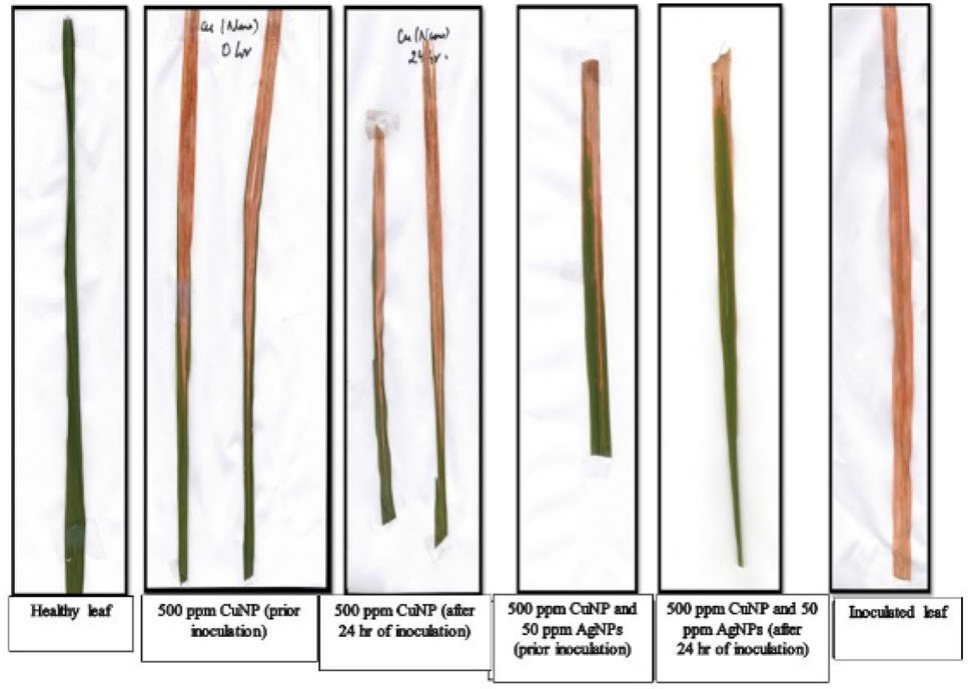
Preliminary field trial data of the nano-composite formulation.

Preliminary field trial of the Cu and Ag nano-composite formulation were also carried out to evaluate the efficacy of the nano-composite formulation in the field condition in cv. TN-1 of rice. Single application of copper nanoparticle was not very effective, but when combined with silver nano-formulation, effectiveness increased significantly (Fig. 6). This study suggests that copper and silver nano-composites were used against the bacterial blight of rice more successfully than the individual nanoparticle.

### In silico studies

**Figure 7:**
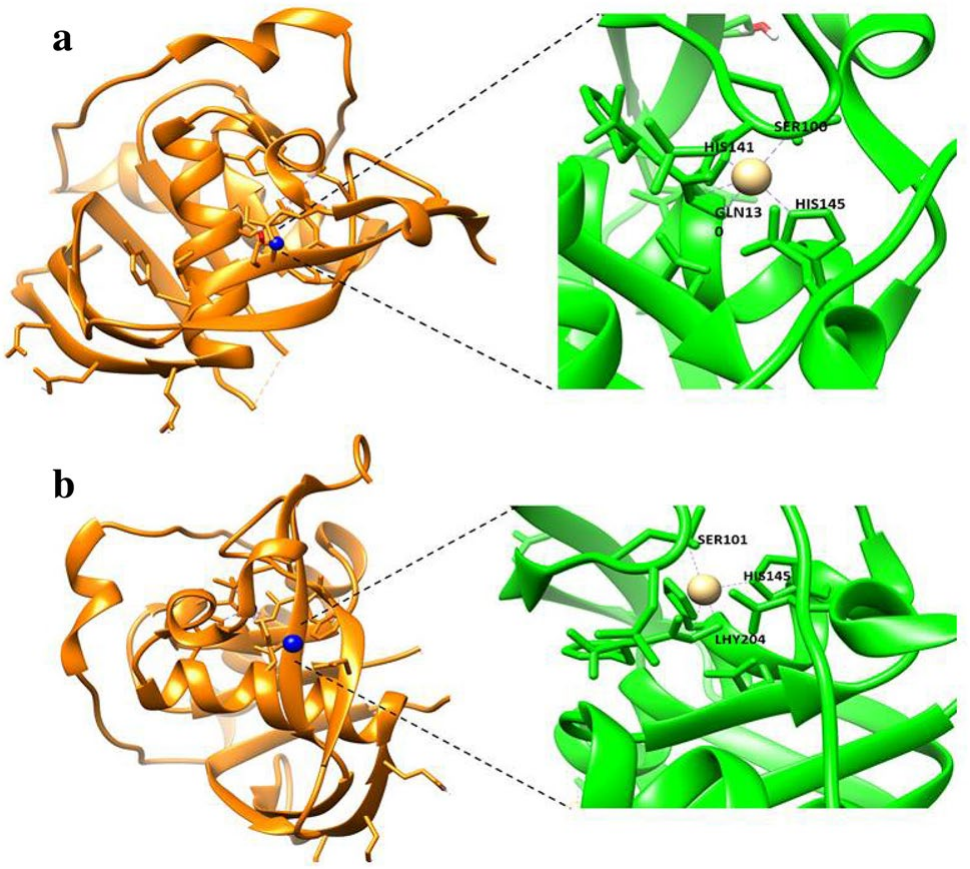
(**a**, **b**) Complex structure of Peptide deformylase-nanoparticle. (**a**) Cu nanoparticle, (**b**) Ag nanoparticle. The protein and nanoparticle are represented in golden yellow and blue colour, respectively.

**Figure 8:**
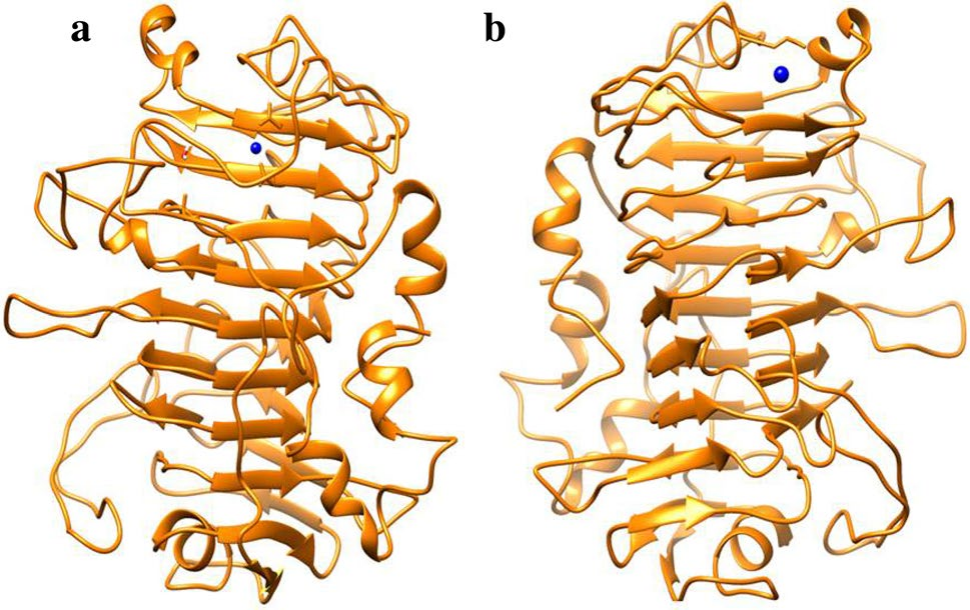
(**a**, **b**) Complex structure of Pectate lyase-nanoparticle. (**a**) Cu nanoparticle, (**b**) Ag nanoparticle. The protein and nanoparticle are represented in golden yellow and blue colour, respectively.

Bacterial leaf blight of rice caused by *Xanthomonas oryzae* pv*. oryzae (Xoo)* and pathogenic fungus *Rhizoctonia solani* that is the cause of sheath blight disease in rice. Therefore, in silico studies were performed on two disease-causing proteins (peptide deformylase and pectate lyase) of bacterial and pathogenic sources^42,47^. Due to the unavailability of the molecular 3D structure of pectate lyase, the Modeller software generated five models of the given input sequence (GenBank: KAF8761373.1) with DOPE scores. The best model (pectate.B99990002.pdb) was selected for further studies depending on the lowest DOPE score (− 36,692.70313), which indicates the free energy and stability of modelled structure (Table 3). Again, Ramachandran Plot was also analysed to validate the model integrity with the value of percentage residues in allowed and disallowed regions (Table 3). The total percentage of allowed region (most favoured and additional allowed) is 92.3% that can be considered as a good model^48^. The docking studies require 3D structure of both protein and nanoparticles (Cu and Ag) that were given as input files in PatchDock server. The server applied shape complimentary algorithm for better docking results. In the present study, the active site region was also provided to perform local docking that can enhance the docking efficiency (Table 4). At the end of the docking, unaccepted complexes were discarded and best 20 results were listed (Table 5) based on geometric shape complimentary score^49^. The result shows that Cu and Ag nanoparticles perform docking with the targeted proteins (Figs. 7a,b, 8a,b). Depending on the score, both proteins generated better complexes with Cu nanoparticles than with silver nanoparticles. The interaction area (Table 4) also indicates that Cu can make more stable complex with proteins and can be more beneficial in inhibiting the disease progression than Ag.

**Table 3:** Modelled structure of pectate lyase using Modeler software.

Model Name	DOPE score	Most Favoured region	Additional allowed regions	Disallowed Region
pectate.B99990001.pdb	− 36,574.71875			
pectate.B99990002.pdb	− **36,692.70313**	**77.3%**	**15.0%**	**2.5%**
pectate.B99990003.pdb	− 35,546.71094			
pectate.B99990004.pdb	− 35,668.56641			
pectate.B99990005.pdb	− 36,358.58984			

Dope score and Ramachandran plot statistics is also provided for the best model (in bold).

**Table 4:** Active site information of targeted proteins, peptide deformylase and pectate lyase through CastP server.

Sl. No	Protein name	Active sites
1	Peptide deformylase	HIS^43^, GLY^44^, VAL^45^, GLY^46^, GLN^51^, TRP^96^, GLU^97^, GLY^98^, LEU^100^, SER^101^, ILE^102^, PRO^103^, GLY^104^, LEU^105^, ARG^106^, ARG^111^, PHE^134^, ARG^137^, VAL^138^, HIS^141^, GLU^142^, ASP^144^, HIS^145^, ARG^149^, LEU^150^, TYR^151^, ASP^164^
2	Pectate lyase	ARG^81^, VAL^83^, ASN^111^, ALA^112^, HIS^113^, PRO^143^, LEU^145^, THR^151^, TRP^152^, ALA^153^, ARG^154^, VAL^155^, ALA^156^, ASP^157^, HIS^159^, ILE^162^, PHE^163^, ASP^164^, ASP^189^, SER^190^, ALA^193^, GLN^194^, GLY^195^, LEU^196^, GLU^197^, THR^198^, HIS^199^, SER^200^, GLY^202^, GLY^203^, LEU^204^, MET^205^, ARG^224^, ASN^225^, VAL^228^, LYS^229^, GLY^230^, THR^231^, ASN^232^, PHE^234^, ASN^237^, VAL^238^, VAL^239^, ASN^241^, SER^253^, ARG^279^, GKY^280^, ASN^281^

**Table 5:** Patch Dock result of protein-nanoparticle docking.

Protein name	Nanoparticle	Score	Area	ACE	Transformation
Peptide deformylase	Cu	1954	225.80	− 10.51	− 2.89 0.94 1.82 23.86 − 15.97 − 1.97
		1820	213.50	− 5.93	2.82 0.24 − 0.88 23.08 − 16.24 − 1.43
		1748	210.20	− 11.16	1.01 − 1.06 3.02 19.58 − 10.35 − 2.63
		1740	208.00	− 4.29	− 1.97 0.40 − 3.13 19.89 − 11.79 − 2.52
	Ag	424	61.30	0.00	0.52 0.74 2.77 18.97 − 7.91 − 9.85
		420	57.80	0.00	0.58 0.14 0.00 18.02 − 11.33 − 0.84
		358	48.40	0.00	2.16 − 0.21 2.31 18.52 − 24.43 14.61
		348	42.40	0.00	2.91 0.16 2.08 18.09 − 18.19 3.88
Pectate lyase	Cu	2242	255.10	− 31.81	2.06 0.84 − 1.66 1.06 − 0.12 52.62
		2192	252.40	− 29.24	− 2.24 − 0.26 0.48 0.69 0.32 52.26
		2158	242.20	− 32.46	− 1.15 0.27 1.51 5.66 − 5.96 57.49
		2140	240.90	− 32.91	2.20 − 0.30 − 1.74 5.88 − 5.53 56.77
	Ag	466	59.90	0.00	− 2.25 − 0.95 − 1.25 2.14 8.92 54.14
		394	48.40	0.00	− 1.37 − 0.29 1.68 6.12 − 6.92 60.07
		386	5050	0.00	0.43 0.61 2.60 18.93 23.08 51.57
		380	49.70	0.00	− 2.89 0.10 − 2.79 9.59 − 6.19 51.54

## Discussion

The bacterial blight and sheath blight diseases are among the most devastating diseases of rice crop causing huge amount of yield loss. Yield losses caused by bacterial blight disease range from 20 to 30% and can be as high as 50% in some areas^50^. Similarly, the yield loss due to the sheath blight disease may also go up to 50%^47,51^. Employing resistant cultivars is one of the options that could be utilized for minimizing yield losses. However, while a number of bacterial blight resistant cultivars have been developed, variety showing high degree of resistance to sheath blight disease is still to be identified. Moreover, even if a number a cultivar resistant to bacterial blight disease have been developed, availability of seed as well as durability of resistance is a cause of concern. On the other hand, a number of pesticides have been used to manage these diseases. However, development of resistance to these pesticides is also a concern. Therefore, newer chemical agents for managing the disease are being looked for. In this context, in the present study, the potential of copper and silver nanoparticle composites was evaluated against *X. oryzae* pv*. oryzae* and *R. solani*.

In the current study, a chemical reduction approach was used to create metallic Copper nanoparticles by chemically reducing Cu^2+^ ions in an aqueous media with ascorbic acid. These metallic Copper nanoparticles were then stabilized using gum arabic. Due to its poor reducing strength, ascorbic acid was employed as a reducing agent. As a result, the reaction driving force is modest and Cu nanoparticle aggregation is difficult. The chemical reduction method for synthesis of copper nanoparticle was also supported by earlier study^52,53^.

Similarly, a biological synthesis method was used for the synthesis of silver nanoparticle. The formation of silver nanoparticle was confirmed by the appearance of yellow–brown color in the reaction mixture. These findings are in tune with earlier leaf extract based AgNPs syntheses carried out in different leaf extract^45,46,54,55^.

The UV–Vis spectrums were recorded for both CuNPs and AgNPs nanoparticles that showed characteristic peaks at 575 nm and 415–440 range, respectively because of the surface plasmon resonance. The surface Plasmon Resonance (SPR) effect is characteristic phenomenon of the small metal nanoparticles due to its absorption of visible electromagnetic waves by the collective oscillation of conduction electrons at the surface^43^.

The synthesized nanoparticles were further characterized by electron microscopy that showed size of both CuNPs was below 100 nm. The EDX spectrum of CuNPs showed the presence of pure elemental copper indicating the purity of the nanoparticle. The EDX data of the synthesized CuNPs also corroborated with the earlier reports^26,56^. The size of silver nanoparticles was determined by TEM that showed it was lower than 100 nm and the DLS analysis showed that it was largely mono-dispersed in nature which are comparable to those reported by Adak et al.^57^. The size, surface charge and PDI of the prepared nanocomposite of CuNPs and AgNPs were measured by DLS and found that size of the prepared nanocomposite were increased moderately as compared to both nanoparticles. The size of the nanocomposite were governed by size of the CuNPs as the main component of the nanocomposite. The surface charge or zeta potential and PDI of the nanocomposite were improved than both the component nanoparticles (CuNPs and AgNPs). The combined effect of the stabilizing agent present in the component nanoparticles were the probable reason behind the improvement in the zeta potential as well as PDI of the nanocomposite. However, the precise mechanism governing the size and growth of the AgCu-NP with various silver and copper content is unknown and requires further research.

The antibacterial activity of CuNPs and CuNPs + AgNPs composites were evaluated against *X. oryzae* pv. *oryzae* in both in vitro and in vivo conditions. In the in vitro study carried out using the well diffusion method, the diameter of zone of inhibition increased with increase in concentrations. The zone of inhibition was more in case of CuNPs + AgNPs composite as compared to the CuNPs indicating the synergistic effect of CuNPs + AgNPs. In the present study, we have reported the antibacterial activity at concentration 250 ppm CuNPs and more. Other lower concentrations had also been used; however, the zone of inhibitions was not clearly visible. Similar to the present study, a recent work have reported that they could not observe any bacterial growth inhibition at or below the concentration of 0.8 μg/ml for CuNPs against *X. oryzae* pv. *oryzae*^58^. Similar to in vitro study, the CuNPs-AgNPs composites were found to be more effective than the CuNPs alone in the in vivo study also.

Similar to the antibacterial activity, the antifungal activity of the nanoparticles against *Rhizoctonia solani* was found to be increased with higher concentration. The inhibitory potential of CuNPs + AgNPs was found to be higher than the singly used CuNPs. It has also reported the antifungal activity of both copper and silver nanoparticles against *R. solani*^59^*.* However, the study have also reported the antifungal activity of individual nanoparticle^59^. To our knowledge, this is perhaps the first report on the antibacterial and antifungal activities of CuNPs + AgNPs composite. Nanoparticles have been reported to be effective tools for managing plant diseases even in other crops in recent times^60,61^.

The antimicrobial activities of metal nanoparticles have been attributed to their ability to generate reactive oxygen species^58,62^. However, the exact mechanism of the antimicrobial activity still needs to be unraveled. Docking analysis was carried out using proteins associated with virulence; peptide deformylase of *X. oryzae* pv. *oryzae* and pectate lyase of *R. solani*. *X. oryzae* pv. *oryzae* and *R. solani* that are the prime factors causing leaf blight disease in plant bodies and till date they are considered as major threats to rice crop^63,64^. Till date no chemical or pesticide is reported that can eradicate the disease with no toxicity to plant bodies. Therefore, an in-silico approach was taken here to explore the interactions of copper and silver nanoparticles with selected proteins and find any potent cure to the disease^42^. Molecular docking is a process to find the best complex of protein and small molecules (nanoparticle). Docking represents the predominant poses of small molecule with protein’s active site and therefore, can illustrate the interaction mechanism with its binding value^65^. Amino acid residues that interact with Cu and Ag nanoparticles are represented in Fig. 6. It is clear from the close-up view of the interactions that Cu and Ag interacts with HIS145, SER100, SER101 and GLN130 of peptide deformylase. The proteins were found to be interacting well with the metal nanoparticles and the interaction was found to be stronger in case of copper as compared to silver. It is already reported that bonded interaction displays stronger complex formation that also help in screening of small molecules for particular plant disease^66^. Here the docking studies infer that the active site of selected proteins (peptide deformylase and pectate lyase) can act as key area to inhibit the enzymatic activity of disease propagation and is crucial for finding inhibitors for pathogenesis. This shows that these proteins could be potential targets of the nanoparticles and further studies would be required to confirm this.

The metallic nanoparticles are known to have high antimicrobial activity. Among the metals, copper and its compounds are readily used in agriculture especially for managing crop diseases. Thus, the antibacterial and antifungal activity of the CuNPs was tested against the causal agents of two most important diseases of rice plant. Further, inhibitory potential of CuNPs-AgNPs composite was evaluated to see if the ability to manage the disease could be enhanced with addition of AgNPs. Silver is more expensive in comparison to copper and thus singly using AgNPs for managing crop diseases could increase the input cost and thus, the antimicrobial activity of the AgNP was not tested in the present study. The CuNPs-AgNPs composite was found to be having greater ability to inhibit the plant pathogens and further field level studies could be carried out to evaluate their efficacy to manage the diseases.

## Methods

All the chemicals and reagents used in the experiment were of analytical grade. Copper sulphate pentahydrate CuSO_4_,5H_2_O, Ascorbic acid C_6_H_8_O_6_ and Sodium hydroxide NaOH were purchased from Merck and Gum Arabic sample was received from ICAR-CAZRI, Jodhpur and used as received without further purification. De-ionized water was used for all the experiments.

### Synthesis of copper nanoparticles

The Cu nanoparticles were synthesized by chemical reduction with slight modification of the process described earlier^26,67^. Briefly, 50 ml aqueous solution of Copper (II) sulfate pentahydrate (0.1 M) was blended with 120 ml Gum Arabic (1%) with vigorous stirring for 30 min. After that, 50 ml 0.2 M aqueous solution of ascorbic acid was added gently into the reaction mixture under continuous stirring condition. Afterwards, 1 M sodium hydroxide solution (30 ml) was added drop wise into the reaction mixture and heated at 80 °C for 2 h while stirring. The colour of the reaction mixture turned yellow to ochre indicated the formation of copper nanoparticle (CuNPs). Reaction mixture was centrifuged and washed repeatedly with double distilled water to remove unreacted reagents and processed for further characterization.

### Synthesis of silver nanoparticle (AgNPs)

A facile and green method of silver nanoparticle synthesis was standardized applying rice leaf extract with some variation of the method reported earlier^45^. At first, fresh rice leaves were collected, washed thoroughly with distilled water and leaf extract was prepared by boiling 5 g of leaf in 50 ml of distilled water followed by filtration and decantation of the extract. 1 mM silver nitrate (AgNO_3_) solution was mixed with the prepared rice leaf extract in different ratios (0.5%, 1.0%, 2.0% and 5.0%). The pH of the mixture was raised to alkaline range and kept at ambient temperature till the colour of the mixture changed to yellow–brown which confirms the formation of silver nanoparticle.

### Characterization of copper nanoparticle

The synthesis of CuNPs was initially confirmed by measuring the absorbance in the range 200–700 nm in a UV–Vis spectrophotometer (CECIL Model CE 7200, UK). To evaluate the potential functional groups associated in synthetic methods of copper nanoparticles, FT-IR analysis were conducted in IR-Prestige 21, Shimadzu Japan, in the range of 400–4000 cm^−1^ at a resolution of 4 cm^−1^. Small amount of copper nano-powder was taken to make KBr pellet and thereafter were processed for FT-IR study and analysed through inbuilt software. The size and surface morphology of the synthesized CuNP was determined by Field Emission Scanning Electron Microscope (FE-SEM) from Carl Zeiss Sigma 300, Germany combined with focused ion beams. The elemental composition was determined by Energy Dispersive X-ray spectroscopy (EDX) using the same FE-SEM instrument in a particular area of the samples. X-ray diffraction (XRD) of the synthesized CuNPs was performed using a Smart Lab 9 kW Rigaku, Japan, X-ray Diffractometer. In-built software program was used for the assignment of reflections and analysis of the XRD patterns. According to the JCPDS (Joint Committee on Powder Diffraction Standards) database, the broadening of the diffraction peaks corresponding to the most intensive reflections was used to measure the mean size of nanocrystals. The XRD diffraction pattern recorded for nanoparticles was used to calculate the crystallite size using the Scherrer equation:

$${\text{d}} = {\text{K}}\uplambda /{\text{B}}\;\cos\uptheta$$

where K is the Scherrer constant (shape factor, its value is 0.9), k is the X-ray wavelength (K = 0.154 nm), B is the line broadening at half the maximum intensity (FWHM) in radians, h is the Bragg angle, (the position of the diffraction peak maximum) and d is the averaged dimension of crystallites in nanometers.

### Characterization of silver nanoparticle

The synthesized AgNPs was characterized by measuring the absorbance in the range 200–700 nm using UV–Vis spectroscopy (CECIL Model CE 7200, UK). The synthesized AgNPs solutions were diluted with Milli-Q water for the particle size analysis, and the DLS particle size analyser (Model: Litesizer 500; Make: Anton Paar, Austria ) was used to measure the particle size and zeta potential. The size and shape of the synthesized silver nanoparticle samples were characterized using Transmission Electron Microscope (TEM, Model JEOL JEM-1011, Japan). Nanoparticle samples were prepared for electron microscopic analysis on a carbon coated Ni + Pd grid of 400 nm by placing a drop of colloidal solution on it followed by vacuum drying.

### Preparation of nanocomposite of CuNPs and AgNPs and their characterization

The synthesized CuNPs sample were diluted with Mili-Q water to make 5000 ppm CuNP and synthesized AgNP sample were also diluted to make 100 ppm AgNP. Before the preparation of nanocomposite of CuNPs and AgNPs, both the CuNPs and AgNPs were be ultrasonicated (Model: SKL-150D, Make- Ningbo Sjia lab Equipment Co Ltd, China) for 5–7 min for proper dispersion of the CuNPs and AgNPs. For preparation of the nanocomposite CuNPs and AgNPs in the same solution, we have to take AgNPs 1/10th of the concentration of the CuNPs. For preparation 10 ml of the CuNP & AgNP nanocomposite suspension having concentration 500 ppm CuNPs and 50 ppm AgNPs in the solution, for that we have to Add 1 ml of the 5000 ppm CuNP Stock solution and 5 ml of 100 ppm of AgNPs stock solution and rest of the volume was made up by 4 ml of the distilled water and the prepared suspension were again subjected to ultrasonication for 5–7 min for proper dispersion of the nanocomposite. Similarly, the different dilution of CuNPs and AgNPs nanocomposite suspension were prepared. The particle size and zeta potential of the prepared nanocomposite of CuNPs and AgNPs were measured using DLS particle size analyzer (Model: Litesizer 500, Make: Anton Paar, Austria).

### Antibacterial and antifungal activity of nano-composites of CuNPs and AgNPs

The antimicrobial activity of nano-composites of CuNPs and AgNPs were evaluated against the leaf blight causing bacteria *Xanthomonas oryzae* pv. *oryzae* and the sheath blight disease causing pathogenic fungus *Rhizoctonia solani*. One of the virulent strains *Xanthomonas oryzae* pv. *oryzae* was cultured in nutrient agar medium and antimicrobial activity of CuNPs and AgNPs was tested by well diffusion method. Briefly, a 5 mm wide well was created on the bacteria inoculated plate by a borer and 30 µl of nano-composite solution (500 ppm CuNPs + 50 ppm AgNPs or 250 ppm CuNPs + 25 ppm AgNPs) was put into the well. Simultaneously, wells filled with different concentrations of CuNPs (500 ppm/250 ppm) and with distilled water served as controls. The diameters of the zone of inhibition were measured using a measuring scale.

The antifungal activity against *Rhizoctonia solani* was evaluated by poisoned food method. Nanoparticles in different concentrations (CuNPs 50 ppm + 5 ppm AgNPs; CuNPs 100 ppm + 10 ppm AgNPs and CuNPs 150 ppm + 15 ppm AgNPs and CuNPs 50 ppm, 100pmm and 150 ppm) were mixed with the Potato dextrose agar (PDA) medium and one of the virulent strains of *R. solani* (MK790180) was streaked on to the medium in Petri plate. The plates were incubated in an incubator at 27 ± 5 °C. Block of fungal mat of *R. solani* inoculated on medium without any addition of nanoparticles served as the control. The efficacy of the CuNPs + AgNPs mixture was measured in terms of percentage growth inhibition using the following formula:

Percent growth inhibition = (C-T) *100/C, where C = mycelial growth in mm in control, and T = mycelial growth in mm in treatment.

### Net house trials of copper and silver nano-composite formulations against *Xanthomonas oryzae* pv. *oryzae* in TN-1 cultivar of rice

Preliminary net house trial of the Cu and Ag nano-composite formulation was also carried out to evaluate the efficacy of the nano-composite formulation in the field condition in cultivar highly susceptible for BLB i.e., TN-1 cultivar of rice. TN-1 rice cultivars were raised in pots under ideal nutrient and growth conditions for experimentation. For evaluation of the efficacy of Cu and Ag nano-composites, the formulations (500 ppm CuNPs + 50 ppm AgNPs) were sprayed as well as individually CuNPs (500 ppm) on cv. TN-1 cultivar of plants in three replications. Nano-formulations were applied through spraying before the inoculation of the disease and another spray of nano-formulation were applied 24 h after the inoculation. Rice plants were inoculated artificially using the leaf clipping technique, wherein 1–2 cm of the leaf tips were cut using scissors dipped in a 1.0 optical density bacterial suspension of *Xanthomonas oryzae* pv. *oryzae*, and observations were made every 7 days interval. As negative and positive controls, healthy and inoculated cv. TN-1 rice plants, respectively, were maintained.

### In silico studies

Two proteins, Peptide deformylase (*Xanthomonas oryzae* pv. *oryzae*) and Pectate lyase (*Rhizoctonia solani*) were selected to perform the molecular docking and interaction studies with silver and copper nanoparticles. The fasta format sequence of pectate lyase (GenBank: KAF8761373.1) and molecular 3D structure of Peptide deformylase (6IL2) were retrieved from public databases, NCBI (National Center for Biotechnology Information (nih.gov)) and PDB (RCSB PDB: Homepage), respectively. Through the Modeller 10.1 software, five models of pectate lyase were generated^68^ and structure was validated using Ramachandran Plot analysis for further docking studies. The mol file of silver and copper nanoparticles were retrieved from PubChem database (PubChem (nih.gov) and then they were converted to PDB file through Chimera visualization software. To perform site-specific docking, CastP server result was analysed for protein active sites. Online server PatchDock [PatchDock Server (tau.ac.il)] was used for docking studies, where receptor and ligand PDB files were taken as input files. Both the proteins were eventually docked with copper and silver nanoparticles and finally the server provided with the best 20 complex structures for each query.

### Policy statement

This to inform that collection of plant materials is compiled with National guidelines and legislation. This is to declare that permission is taken for use rice leaf, germplasm.

## Conclusion

Current evaluation elucidates a green technology for developing copper and silver nanoparticles and their potent combinatorial role as a composite. Moreover, findings suggest that copper silver nano-composites has dual phenomenon as antibacterial and antifungal candidate in comparison to copper nanoparticles applied in singular dose. Copper and silver composite in nanoform could be applied for developing commercial formulations to manage the devastating rice diseases like bacterial blight and sheath blight to bring better efficacy for Integrated Pest Management system in rice crop.

## Data Availability

The datasets generated during and/or analysed during the current study are available from the corresponding author on reasonable request.
